# Mothers’ Mental State Talk and 3- to 5-Year-Old Children’s Theory of Mind: Their Reciprocal Dynamic Impact

**DOI:** 10.3390/bs14070568

**Published:** 2024-07-05

**Authors:** Hang Liu, Fan Wu, Siying Li, Haiting Zhang

**Affiliations:** Faculty of Education, Northeast Normal University, Changchun 130024, China; liuh974@nenu.edu.cn (H.L.); wufan@nenu.edu.cn (F.W.); zhanghaiting@nenu.edu.cn (H.Z.)

**Keywords:** mental state talk, theory of mind, mother–child reading, false-belief understanding

## Abstract

The development of children’s social function is influenced by the social environment, and children also play an active role in shaping and nurturing this environment. The present study investigated the potential reciprocal dynamic impact of mothers’ mental state talk (MST) and the development of theory of mind (ToM) in children. Using a cross-sectional design, we explored the development of ToM in 3- to 5-year-old children using an unexpected location task, an unexpected content task, an appearance–reality task, and mothers’ MST during mother–child reading. The results showed the following: (1) the mothers’ MST exhibited a shift from a predilection for subjective terms (such as desires) to a preference for objective terms (such as cognitions) as their children matured; (2) the development of the children’s ToM was closely related to their mothers’ MST. The overall MST scores, as well as the different categories and referents used in MST, were closely associated with the children’s ToM development. Notably, at the age of four, a critical period emerged where the correlation between maternal MST and ToM became significantly pronounced. These findings highlight the reciprocal nature of mother–child interactions as a two-way adaptive process.

## 1. Introduction

In social interactions, individuals are constantly faced with the ongoing challenge of understanding the dynamic and covert mental states of others. Individuals who navigate their social environments also gradually acquire the ability to understand the mental states of others during the growth process, including their desires, knowledge, beliefs, emotions, and other internal experiences, and use this information to explain and predict others’ behaviors; this process is referred to as theory of mind (ToM) [1,2]. This ability, which serves as a means for individuals to establish connections with society, is closely associated with various cognitive [3,4] and social functions [5,6,7,8] and is essential for the assimilation of individuals into society and the cultivation of harmonious interpersonal relationships. The factors that contribute to the development of children’s ToM ability can be categorized into two domains: individual maturation and maturation-related cognitive development [1] as internal factors, and environmental suitability as an external factor [9,10]. ToM ability continues to develop during a child’s socialization process, in which the input of their mother has an important external influence [9,10]. Mothers’ mental state talk (MST), which refers to conversations between a child and their mother about mental states such as thoughts, beliefs, and desires [11,12], provides valuable social cues to children and serves as a crucial foundation for the development of ToM ability [13,14,15,16]. The present study built upon these foundations and considered the following question: does a reciprocal dynamic exist between mothers’ influence and children’s maturation in the development of children’s ToM abilities? Regarding this issue, the present study systematically explored two relevant aspects: (1) the characteristics of mothers’ MST during mother–child reading with children of varying ages; (2) the association between mothers’ MST and the development of ToM in children aged 3 to 5 years.

The development of children’s ToM ability is a crucial indication of their progressive maturation in terms of social cognition, which is an essential prerequisite for their successful integration into social life [17]. The ToM abilities of children develop as they mature, which is reflected in their enhanced comprehension of false beliefs. The development of this social function occurs within the first five years of life, during which 3-year-old children acquire an understanding that mental states can vary across individuals [1]. The emergence of false-belief understanding (FBU) in children around the age of 4 years is considered indicative of a significant cognitive breakthrough [18]. However, there appears variation in the age at which children reach key developmental milestones. Previous studies have shown that the attainment of success in false-belief tasks varies among children, with some achieving success as early as the age of 3 years, while others not demonstrating understanding until the age of 6 years [19]. ToM, as a social cognition outcome, is closely associated with the guidance provided by adults during the growth process, which in turn contributes to individual differences in children’s ToM abilities [20]. Interactions between children and adults provide children with ample exposure to MST. For example, reading together allows parents and children to engage in meaningful discussions regarding mental states, such as the thoughts, beliefs, and desires of various characters [21]. This experience plays a crucial role in facilitating children’s comprehension of others’ mental states.

The efficacy of parental MST in enhancing ToM ability in children has been substantiated by numerous empirical studies [11,22,23,24,25], including studies involving exceptional children [26,27,28]. A meta-analysis examined the correlations between child FBU and parental MST, and these associations remained significant even after controlling for individual differences in verbal ability [29]. The effect of parental MST on children’s FBU has been further substantiated by copious evidence from longitudinal studies [12,19,30]. A longitudinal study revealed that individual differences in mothers’ MST to 2-year-old children predicted variations in children’s FBU at the age of 6 years and their strange story scores at the age of 10 years [19]. Another previous study revealed a unique association between early mothers’ MST and both the subsequent development of ToM ability in children and later measures of conflict and cooperation [31].

The utilization of MST commonly involves the expression of specific categories of mental state terms, such as desire terms (e.g., like), emotion terms (e.g., happy), and cognitive terms (e.g., think) [11]. These terms denoting mental states are typically used in conjunction with words referring to a person to represent an individual’s specific mental state [10,14,32]. When combining the categories and referents of MST, one study revealed that parents’ MST regarding their own emotions and desires and others’ cognitions, but not their children’s mental states, significantly influenced their children’s FBU [11]. MST enables parents to explicitly refer to internal states and direct children’s attention toward such states, which are always implicit [16,33]. The aforementioned process of MST has dual effects on children’s development of ToM: first, it facilitates the association between external behaviors and internal mental states in children; second, it enables children to comprehend the mental states of others through different character perspectives. These positive effects serve as a valuable point of reference for the practical guidance of child development.

Studies often focus on developmental changes in children and may inadvertently overlook the reciprocal nature of the caregiver–child influence, where children can also guide their caregivers toward adopting appropriate parenting strategies. The present study further focused on the relationship between the development of ToM ability in children and mothers’ MST. Children aged 3–5 years and their parents were selected for this study because early childhood is an important stage for parents and children to engage in shared reading, as well as for the acquisition and development of psychological theories about children. Based on a cross-sectional design, we investigated the development of ToM in 3-to-5-year-old children using an unexpected location task, an unexpected content task, and an appearance–reality task, a strategy that has demonstrated high reliability and internal consistency [34]. Simultaneously, we analyzed the implementation of MST during mother–child reading. Mothers’ MST was categorized into three dimensions: categories (desires, emotions, and cognitions), referents (mothers’ own, children’s, and story characters’ mental state), and utterance functions (questions and comments). These three dimensions were further classified and combined. Using a fine-grained approach, we explored (1) distinctive variations in maternal MST during interactions with children across different ages; (2) the relationship between using diverse categories and referents and their combination in the maternal MST and the development of children’s ToM abilities.

## 2. Materials and Methods

### 2.1. Participants

A total of 220 children and their mothers in two kindergartens were randomly recruited as participants in Wuxi, Jiangsu Province, China. Seven children who could not complete the experimental tasks were excluded due to delayed language development and limited language ability, ten children were excluded due to the incomplete recording of mother–child reading videos, and nine children were excluded due to the short duration of mother–child reading time, which was less than 3 min. Ultimately, 194 valid mother–child pairs were included. The distribution of the children was as follows: 73 boys, (*M* = 4.52 years, *SD* = 0.89 years); 63 children aged 3 years (28 boys, range = 3.08–3.92 years, *M* = 3.53 years, *SD* = 0.26 years); 65 children aged 4 years (24 boys, range = 4.00–4.92 years, *M* = 4.40 years, *SD* = 0.27 years); and 66 children aged 5 years (21 boys, range = 5.00–6.00 years, *M* = 5.59 years, *SD* = 0.30 years). All participants included in this study were typically developing children. The present study was reviewed and approved by the Ethics Review Committee of the School of Psychology, Northeast Normal University. The mothers were informed about the purpose of the study and provided written consent.

### 2.2. Materials and Procedure

#### 2.2.1. ToM Tasks

In a quiet classroom, a researcher worked together to complete the tests with each child. The researcher sat face-to-face with the child at a table, on which the experimental materials were placed. The average testing time for each child was approximately 5 min. To control the order effect of the tasks, the order of the tasks and questions was balanced among the children, and the same form was used to record the children’s answers. Before the experiment, the researcher played games with the children to help them overcome any sense of unfamiliarity. After the experiment, each child was given a sticker as a reward.

The children’s ToM development level was assessed using the unexpected location, unexpected content, and appearance–reality tasks. The unexpected location task was adapted from Wimmer and Perner [35]. The researcher told the children a story while using props to visually demonstrate the following plot: “Peggy put an eraser in a pencil case and then went out to play. George moved the eraser from the pencil case to a bag.” After listening to the story, the children answered the following four questions: the memory control question “Where did Peggy put the eraser before she went out to play?”; the reality detection question “Where is the eraser now?”; the behavior prediction question “Where should Peggy go to look for the eraser first?”; and the false belief question “Where does Peggy think the eraser is?”.

The unexpected content task was adapted from Gopnik and Astington [36]. The researcher showed a sealed potato chip canister to the child and asked “What do you think is inside this canister?”. After the child answered, the researcher opened the potato chip canister and took out a pencil to show the child what was inside the canister. Then, the researcher resealed the potato chip canister. Subsequently, the child answered three questions: the detection question “What do you think is inside the canister now?”, the self false-belief question “What did you think was inside the canister before it was opened?”, and the others’ false-belief question, “If XXX (a familiar playmate for the child) had never opened this canister, what would he/she think was inside the canister when he/she saw it?”.

The appearance–reality task was adapted from Flavell et al. [37]. The researcher showed the child an object that looked like an apple but was actually made of plastic. The child needed to answer two questions: the appearance question “What does this object look like to you now when you look at it with your eyes?” and the reality question “What is this object actually made of?”.

#### 2.2.2. Mother–Child Reading Task

The mother–child reading task was adopted to collect data on the mothers’ MST and has been used in previous studies to assess mothers’ MST [14,38]. Before the task began, the researcher provided each mother with a 32-page wordless picture book titled “The Snowman”, which was published by Tomorrow Publishing House in 2020. This wordless picture book depicts a wonderful story involving a little boy and a snowman he builds. The length and cognitive level of the story are suitable for preschool children, and the story is rich in mental states.

**Figure 1:**
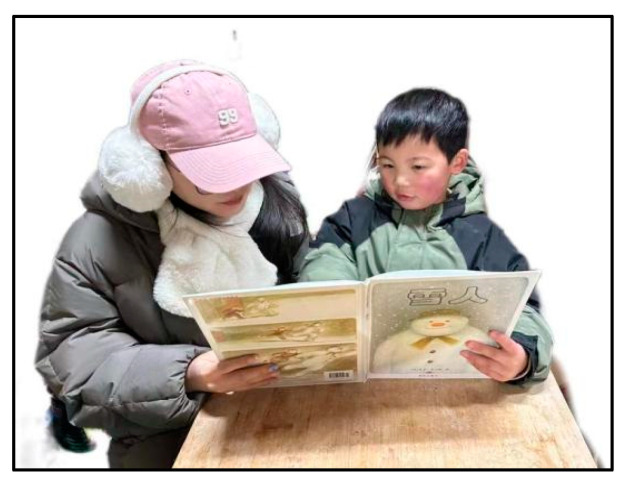
Image of mother–child reading video recording.

Mothers and children read together in a familiar home environment, and the process of reading was recorded (Figure 1). The mothers were asked to maintain a reading style consistent with their daily home reading habits while reading the entire picture book. The mothers recorded the entire reading process using a mobile phone, a camera, or another device. Both the mother and the child were visible from the waist up in the video. The recording device was placed approximately one meter in front of the mother and child to facilitate the observation of their facial expressions and body positions. Prior to video recording, the researcher provided comprehensive instructions and training to the mothers. These instructions covered the basic operation of the recording equipment, the selection and optimization of the recording environment, video composition, and other professional and technical details to ensure the authenticity and high quality of the video content. After the completion of the reading session, the mother sent the recorded video (in mp4 format) to the researcher’s designated email address. In total, 194 valid videos were collected, with each video limited to under 20 min in length, resulting in a total duration of 1636.25 min (*M* = 8.06 min, *SD* = 4.16 min).

### 2.3. Scoring and Coding

#### 2.3.1. ToM Tasks

In the unexpected location task, 1 point is awarded for the correct response to both the behavior prediction question and the false belief question, and 0 points are awarded for an incorrect response. In the unexpected content task, 1 point is awarded for the correct response to both the self false-belief question and the others’ false-belief question, and 0 points are awarded for an incorrect response. In the appearance–reality task, 1 point is awarded for the correct response to both the appearance question and the reality question, and 0 points are awarded for an incorrect response. The total score for the ToM tasks ranges from 0 to 6 points.

#### 2.3.2. Mother–Child Reading Task

The mothers’ MST during the mother–child reading task was transcribed and coded. The content of the coding consisted of two parts: the reading of the picture book and the discussion about the content of the picture book after reading, which lasted from the time the mother started to read the cover of the picture book until the time the mother closed the book. Repetitive, meaningless speech or exclamation words (such as gasps or sighs) were excluded from the transcription. The children’s speech was not transcribed or coded, as their speech frequency was low, and this study focused more on the mothers’ MST. The transcripts of the mother–child reading were coded in Standard Chinese. Two researchers created an English coding scheme based on previous research [11,39], reviewed the transcripts to identify Standard Chinese terms that matched the terms in the English scheme, and added unrecorded mental state terms to create a preliminary coding scheme for Standard Chinese mental states. After reviewing and evaluating the preliminary scheme, they created the final coding scheme. During the coding process, the two coders underwent systematic training, which included a thorough review of the coding guidelines, analysis and discussion of example videos, and several simulated coding exercises. This was done to ensure that they had a clear and consistent understanding of the coding criteria. The two coders coded independently over a five-day period.

The mothers’ MST was coded at two levels: primary coding and secondary coding. The primary coding of the mothers’ MST was conducted based on three dimensions: categories, referents, and utterance functions (Table 1). The referents included mothers, children, and story characters. The utterance functions consisted of questions (open-ended and closed questions) and comments (declarative sentences and imperative sentences).

The total number of utterances under each category constituted the mothers’ scores in that category, and the coding among these three categories was mutually exclusive. Upon completion, the kappa coefficient was used to calculate the coding reliability, which was 0.963 for the categories, 0.979 for the referents, and 0.978 for the utterance functions, indicating good coding consistency.

The secondary coding was composed of the cross combination of three variables, generating 18 new variables.

### 2.4. Data Analysis

In this study, we conducted the following statistical analyses on the survey data using IBM SPSS 26.0 and Mplus 8.3: a correlation analysis, a repeated-measures analysis of variance (ANOVA), a confirmatory factor analysis, a path analysis, and a moderation analysis.

## 3. Results

### 3.1. Preliminary Analysis

Table 2 shows the descriptive statistics for all variables of the study. The values of all variables of the MST were standardized. Table 3 shows the results of the correlation analysis of all variables whose values did not correspond to a normal distribution, with the exception of gender, which was correlated using Spearman’s method.

### 3.2. Characteristics of Mothers’ MST

To examine the differences between the various dimensions of mothers’ MST, a 3 (categories: desires, emotions, cognitions) × 3 (referents: mothers’ own, children’s, story characters’ mental state) × 2 (utterance functions: questions, comments) repeated-measures analysis of variance (ANOVA) was performed with the frequency of standardized MST as the dependent variable. After controlling for mothers’ education and children’s age, the results showed a significant main effect of categories (*F*(2, 190) = 10.136, *p* < 0.001, partial *η_p_*^2^ = 0.096). Further post-hoc tests revealed that cognition terms were significantly more frequent than emotion terms (*M*_cognition–emotion_ = 1.606, *p* < 0.05) and desire terms (*M*_cognition–desire_ = 1.904, *p* < 0.05), and emotion terms more frequent than desire terms (*M*_emotion–desire_ = 0.298, *p* < 0.05). The main effects of referents (*F*(2, 190) = 1.329, *p* = 0.267, partial *η_p_*^2^ = 0.014) and utterance functions (*F*(1, 191) = 0.600, *p* = 0440, partial *η_p_*^2^ = 0.003) were not significant. In addition, the interaction between categories and referents was not significant (*F*(4, 188) = 0.946, *p* = 0.438, partial *η_p_*^2^ = 0.020); the interaction between categories and utterance functions was not significant (*F*(2, 190) = 1.457, *p* = 0.236, partial *η_p_*^2^ = 0.015); and the interaction between referents and utterance functions was not significant (*F*(2, 382) = 0.581, *p* = 0.56, partial *η_p_*^2^ = 0.003), nor was the interaction effect between categories, referents, and utterance functions (*F*(4, 188) = 0.405, *p* = 0.805, partial *η_p_*^2^ = 0.009).

### 3.3. Association between Mothers’ MST and the Development of Children’s ToM

To investigate the relationship between mothers’ MST and children’s ToM, this study first conducted a confirmatory factor analysis to determine the factor structure of ToM. As the model was a saturated model, i.e., all parameters to be estimated were exactly equal to the elements in the covariance matrix with zero degrees of freedom, the fit indices were no longer estimated, and only the path coefficients were considered. All items loaded significantly, with standardized factor loading coefficients ranging from 0.406 to 0.657. In our model, we included the latent factors of ToM as outcome variables.

As previous studies have found that children’s age and SES have an impact on the development of children’s ToM, in the present study children’s age and mothers’ education were included as covariates in the model. All variables of MST were included in the model, and it was found that the model fitted well, as evidenced by the indices *χ*^2^/*df* = 1.342, CFI = 0.920, RMSEA = 0.042, and SRMR = 0.052, with only children’s age and total frequency of MST significantly associated with children’s ToM (*p* < 0.001).

Due to the small sample size, it was not possible to create an overall model that included MST‘s categories, referents, and utterance functions [20]. Therefore, we estimated three separate models categorized by referents. First, we tested the effect of referring to mothers’ own mental state. The model had a good fit, as evidenced by the indices *χ*^2^/*df* = 1.765, CFI = 0.917, RMSEA = 0.063, and SRMR = 0.054, showing that mothers’ cognition comments were significantly associated with children’s ToM. Second, we tested the effects of referring to children’s mental state. The model fitted the data adequately, as evidenced by the indices *χ*^2^/*df* = 1.380, CFI = 0.954, RMSEA = 0.044, and SRMR = 0.050, and in the model, children’s emotion comments, children’s cognition questions, and children’s ToM were significantly correlated. Finally, we tested the effect of referring to the mental state of the story characters. The model fitted well, as evidenced by following indices *χ*^2^/*df* = 1.513, CFI = 0.938, RMSEA = 0.051, and SRMR = 0.052, and no factors were significantly related to children’s ToM in the model.

**Figure 2:**
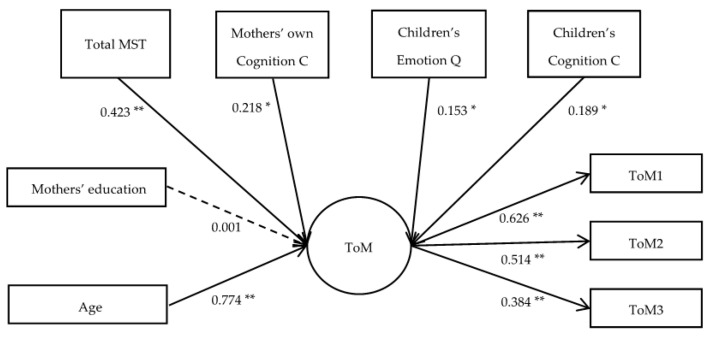
Structural equation explaining the individual differences in children’s ToM. Note. ToM1: unexpected location task; ToM2: unexpected content task; ToM3: appearance–reality task; SC: story characters; Q: questions; C: comments; * *p* < 0.05, ** *p* < 0.01.

To obtain a single simplified model, we estimated a final model that included only the MST variables that were significantly associated with children’s ToM in the previous models (i.e., total MST, mothers’ cognition comments, children’s emotion comments, and children’s cognition questions). The final model fitted well, as evidenced by the indices *χ*^2^/*df* = 1.760, CFI = 0.949, RMSEA = 0.063, and SRMR = 0.054, in which total MST, mothers’ cognition comments, children’s emotion comments, and children’s cognition questions were all significantly correlated with children’s ToM. The final results of the model are shown in Figure 2.

Finally, the moderating role of age was further analyzed in this study on the basis of the overall model. Using a multi-group analysis, the effects of mothers’ total scores for MST, mothers’ cognition comments, children’s emotion comments, and children’s cognition questions on ToM were compared across the three age groups, which differed significantly. A positive effect was observed between mothers’ total scores for MST and children’s ToM (B = −0.730, t = −2.727, *p* < 0.05), with significant differences in the effect among the three groups. Further analysis revealed that the effect was significantly stronger in the 4-year-old group compared to the 5-year-old group (B = 0.433, t = 2.150, *p* < 0.05), while no significant differences were observed between the 3-year-old and the 4-year-old groups, or between the 3-year-old and the 5-year-old groups.

## 4. Discussion

The development of children’s social functioning is continuously shaped by the social environment. Throughout this process, children gradually acquire the ability to discern and integrate subjective and objective information, as well as interpret social cues. This social environment is often actively created by their caregivers, with the mother’s role being of utmost importance [40]. The present study focused on the relationship between the development of ToM in children and mothers’ MST. By using the unexpected location task, unexpected content task, and appearance–reality task, we investigated the development of ToM in children aged 3 to 5 years. Mothers’ MST was assessed during mother–child reading. We found a reciprocal dynamic between mothers and children: the MST strategy employed by the mothers evolved in parallel with the developmental progression of their children and exhibited strong associations with the development of children’s ToM abilities.

### 4.1. Characteristics of Mothers’ MST Change with the Development of Children

This study investigated the use of MST by mothers of children aged 3–5 years during picture book reading. We first categorized MST into three dimensions: categories, referents, and utterance functions. These three dimensions were further classified and combined. This fine-grained approach contributed to exploring the pattern of mothers’ MST among children aged 3 to 5 years. The results revealed a significantly greater frequency of the use of cognition terms than of desire terms and emotion terms by the mothers during interactions with their children. Additionally, the mothers exhibited a significantly greater utilization of emotion terms than of desire terms. This pattern may be associated with the necessity to foster children’s progressively evolving ToM abilities.

Across different stages of children’s development, we observed variations in the way the mothers interacted with their children. In the correlation analysis, we found significant negative correlations between the ages of the children and the frequency of the mothers’ comments on children’s desires as well as questions about children’s emotions. This finding suggests that mothers tend to discuss desire and emotion more frequently with young children, which facilitates their understanding of mental states [10]. The concepts of desire and emotion, corresponding to a lucid and uncomplicated mental state, are more readily comprehensible for young children. These concepts emphasize an individual’s subjective volition while exhibiting limited interaction with the objective world. However, as children grow older, mothers shift toward using less these kinds of terms of mental states, potentially catering to the development of abstract thinking in their children. Our results showed that the utilization of cognitive terms by mothers tends to increase as their children grow older. Compared with mental states with external cues such as desires and emotions, cognitive states are more likely to make children realize the complexity and implicity of mental states [39]. Talking to children about cognitive states based on their understanding of mental states can facilitate their development of cognitive representation in language, which is essential for their later growth and development [28]. This change in mothers’ MST reflects the reciprocal influence between mothers and children during their interactions; mothers regard children as mental individuals with subjective initiative [41], thereby adapting their parenting styles according to their children’s development.

### 4.2. Association between Mothers’ MST and Children’s ToM Development

The present study employed a cross-sectional design to investigate the developmental characteristics of ToM in 3-to-5-year-old children. The findings of the correlation analysis indicated that there were age-related variations in the progression of ToM among children aged 3–5 years, with a gradual increase observed across all tasks as they grew older. These findings are consistent with previous research: children begin to exhibit some degree of ToM at the age of 3 years, a critical ToM development period occurs at approximately 4 years of age, and children aged 5 to 6 years start to acquire ToM [36,42,43,44].

The relationship between mothers’ MST and the development of children’s ToM was another focal point of this study. The results of the correlation analysis and confirmatory factor analysis showed that the mothers who displayed elevated rates of talk about mental state had children who displayed better abilities of ToM. Furthermore, many terms of mothers’ MST were significantly positively correlated with children’s ToM scores. These findings are consistent with previous research findings demonstrating a significant correlation between mothers’ MST during mother–child reading and proficiency in false-belief tasks among children [13]. After establishing a clear and robust correlation between MST and the development of children’s ToM, we conducted a moderating-effect analysis and found variations in this correlation across different age groups. The result demonstrated that the link between MST and children’s ToM was significantly stronger in the 4-year-old group than in the 5-year-old group. This finding offers a crucial indication for our educational strategy. The age of 4 years is pivotal in the development of children’s theoretical psychological abilities and also presents an optimal timeframe for educators to provide effective educational support.

As to the diverse categories, our findings showed that using more mental state terms about emotion and cognition is related to the development of ToM abilities in children. The beneficial impact of cognition terms in MST has been consistently replicated across numerous studies [19,45]. One study found that mothers’ mental state talk about cognition terms in relation to their 2-year-old children predicted their ToM development over eight years, even after accounting for children’s verbal ability [19]. Further, the combinative use of cognition and comment in interactions can help establish a connection between others’ mental states and enable children to infer and assess their mental states from alternative perspectives, thereby facilitating their comprehension of others’ mental states from diverse perspectives. As to the terms of emotion, in our study, inquiring about the emotional state of younger children could effectively sustain their attention, facilitate their engagement with the narrative context, and assist mothers in strategizing the progression of parent–child reading. Previous studies found that mothers’ references to emotion and an emotion causal explanatory language predicted children’s performance in emotion understanding, and fathers’ use of a causal explanatory language referring to emotions predicted children’s ToM abilities [17].

## 5. Conclusions, Limitations, and Future Directions

The study’s findings and conclusions should be considered in light of its limitations. The first concern is the measurement of ToM. In the current study, the ability of ToM was perceived as integrated and was measured through three classical tasks that have demonstrated high reliability and internal consistency. However, the reasoning of ToM is extensive, encompassing a diverse range of concepts and competencies. To explore the relationship between MST and the different aspects of children’s ToM abilities, the multifacetedness of ToM can be captured by measuring tools that can assess ToM ability across levels of complexity and various aspects in future works (such as the Theory of Mind Task Battery [46,47]). Another shortcoming that should be considered is that the findings from this cross-sectional study need to be further consolidated through longitudinal studies. The inclusion of a comprehensive longitudinal design in future research would contribute to understanding the reciprocal dynamic impact between mothers and their children.

In summary, this study interpreted the relationship between mothers’ MST and children’s ToM development from two perspectives and explored their reciprocal dynamic impact reaching the following conclusions: (1) mothers exhibited a shift in their MST from a predilection for subjective terms to a preference for objective terms as their children matured; (2) the frequency of mothers’ MST was closely associated with their children’s development of ToM. At the age of four, a critical period emerged where the correlation between mothers’ MST and ToM became significantly pronounced. These findings increase our understanding of the reciprocal nature of mother–child interactions.

## Figures and Tables

**Table 1:** Primary coding standards for MST.

Variable	Implication	Example
Categories		
Desires	This includes language related to wishes and desires (such as “want”, “desire”, “need”, “like”, “prefer”, “aspire”, “dream”, “hope”, etc.).	The snowman reached out his hand, wanting to touch the light inside the television.
Emotions	This encompasses language associated with emotional states (like “happy”, “sad”, “grieved”, “depressed”, “worried”, “afraid”, “proud”, “satisfied”, etc.).	The snowman was extremely delighted to see these things.
Cognitions	This refers to language related to thoughts or cognitions (such as “think”, “know”, “feel”, “believe”, “trust”, “understand”, “pretend”, “forget”, “remember”, “guess”, “imagine”, “ponder”, etc.).	What do you think he would say to the snowman at this moment?
Referents		
Children	The target object referred to in the MST is the child.	What would you like to call him?
Mothers	The target object referred to in the MST is the mother.	Mom thought he was missing a nose.
Story Characters	The target objects referred to in the MST are characters in the book, i.e., a little boy and a snowman.	The little boy wanted to build a snowman.
Utterance functions		
Questions	Questions include both open and closed questions.	What do you think the snowman is lacking?
Comments	Comments encompass declarative sentences and imperative sentences.	The little boy was quite worried about the snowman.

**Table 2:** Descriptive statistics for all variables (*M* ± *SD*).

Variable	*M*	*SD*	Range
1. Age (year)	4.02	0.82	3–5
2. Gender	37.6% (boy)	–	–
3. Mothers’ education	3.77	0.75	–
4. Total ToM	3.38	1.3	0–6
5. ToM1	0.8	0.82	0–2
6. ToM2	0.69	0.62	0–2
7. ToM3	1.9	0.3	0–2
8. Total MST (hourly)	3.38	1.3	1–6
9. Mothers’ own Desire Q (hourly)	0.05	0.4	0–3.57
10. Mothers’ own Desire C (hourly)	0.09	0.86	0–9.70
11. Mothers’ own Emotion Q (hourly)	0.03	0.31	0–3.11
12. Mothers’ own Emotion C (hourly)	0.04	0.41	0–4.41
13. Mothers’ own Cognition Q (hourly)	0.24	1.16	0–9.86
14. Mothers’ own Cognition C (hourly)	1.1	3.07	0–21.85
15. Children’s Desire Q (hourly)	1.18	6.15	0–77.84
16. Children’s Desire C (hourly)	0.4	1.99	0–15.58
17. Children’s Emotion Q (hourly)	2	4.87	0–31.03
18. Children’s Emotion C (hourly)	1.03	3.13	0–22.31
19. Children’s Cognition Q (hourly)	6.44	11.54	0–63.05
20. Children’s Cognition C (hourly)	1.88	4.37	0–30.11
21. SC Desire Q (hourly)	2.56	5.77	0–46.43
22. SC Desire C (hourly)	10.23	12.23	0–66.67
23. SC Emotion Q (hourly)	12.56	16.08	0–111.86
24. SC Emotion C (hourly)	38.99	32.5	0–201.60
25. SC Cognition Q (hourly)	6.5	9.84	0–60.50
26. SC Cognition C (hourly)	16.81	18.87	0–130.91

Note. ToM1: unexpected location task; ToM2: unexpected content task; ToM3: appearance–reality task; SC: story characters; Q: questions; C: comments.

**Table 3:** Correlation between all variables.

	1	2	3	4	5	6	7	8	9	10	11	12	13	14	15	16	17	18	19	20	21	22	23	24	25	26
1. Age (year)	1																									
2. Gender	0.078	1																								
3. Mothers’ education	0.030	0.011	1																							
4. Total ToM	0.613 **	0.074	−0.045	1																						
5. ToM1	0.525 **	0.102	−0.112	0.841 **	1																					
6. ToM2	0.357 **	0.001	0.022	0.740 **	0.344 **	1																				
7. ToM3	0.400 **	0.087	0.095	0.478 **	0.204 **	0.276 **	1																			
8. Total MST (hourly)	−0.033	0.033	−0.017	0.436 **	0.317 **	0.430 **	0.159 *	1																		
9. Mothers’ own Desire Q (hourly)	−0.054	0.097	−0.098	0.115	0.088	0.125	0.042	0.213 **	1																	
10. Mothers’ own Desire C (hourly)	−0.127	−0.027	0.035	0.012	−0.034	0.062	0.035	0.147 *	0.403 **	1																
11. Mothers’ own Emotion Q (hourly)	−0.002	0.079	−0.138	0.114	0.089	0.122	0.035	0.175 *	0.810 **	−0.010	1															
12. Mothers’ own Emotion C (hourly)	−0.065	−0.027	−0.138	0.093	0.089	0.062	0.035	0.162 *	−0.013	−0.010	−0.010	1														
13. Mothers’ own Cognition Q (hourly)	−0.032	0.069	−0.067	0.138	0.195 **	−0.015	0.075	0.288 **	0.163 *	−0.023	0.211 **	−0.023	1													
14. Mothers’ own Cognition C (hourly)	0.071	0.032	−0.073	0.261 **	0.222 **	0.228 **	0.054	0.363 **	0.158 *	−0.043	0.216 **	0.110	0.217 **	1												
15. Children’s Desire Q (hourly)	−0.107	0.145 *	−0.147 *	0.131	0.081	0.159 *	0.053	0.336 **	0.270 **	0.154 *	0.157 *	0.130	0.086	0.152 *	1											
16. Children’s Desire C (hourly)	−0.148 *	0.085	0.079	−0.097	−0.119	−0.042	−0.001	0.086	0.165 *	0.209 **	−0.024	−0.024	−0.051	0.068	0.012	1										
17. Children’s Emotion Q (hourly)	−0.141 *	−0.028	−0.083	0.062	0.036	0.110	−0.036	0.317 **	0.119	0.053	0.066	0.216 **	0.274 **	0.189 **	0.254 **	−0.021	1									
18. Children’s Emotion C (hourly)	−0.031	−0.034	−0.086	0.128	0.094	0.115	0.014	0.268 **	0.089	−0.038	0.129	−0.038	0.054	0.172 *	0.098	0.175 *	0.138	1								
19. Children’s Cognition Q (hourly)	0.146 *	0.014	−0.048	0.375 **	0.226 **	0.380 **	0.182 *	0.527 **	0.178 *	0.097	0.173 *	0.120	0.097	0.328 **	0.239 **	−0.018	0.226 **	0.175 *	1							
20. Children’s Cognition C (hourly)	0.144 *	−0.027	−0.145 *	0.245 **	0.223 **	0.178*	0.038	0.233 **	0.020	−0.051	0.050	−0.051	0.189 **	0.341 **	0.089	−0.069	0.028	0.382 **	0.154 *	1						
21. SC Desire Q (hourly)	−0.099	0.086	−0.002	0.085	0.062	0.103	0.002	0.358 **	0.121	0.086	0.030	0.208 **	0.201 **	0.066	0.229 **	0.206 **	0.059	−0.023	0.123	0.060	1					
22. SC Desire C (hourly)	−0.105	−0.008	−0.009	0.151 *	0.166 *	0.145 *	−0.003	0.363 **	0.073	−0.054	0.089	0.101	0.041	0.049	0.089	0.031	0.116	−0.026	0.093	0.013	0.221 **	1				
23. SC Emotion Q (hourly)	−0.06	0.024	−0.082	0.037	−0.017	0.068	0.011	0.273 **	0.099	0.054	0.095	0.108	0.040	0.104	0.066	0.085	0.041	0.165 *	0.155 *	0.131	0.320 **	−0.034	1			
24. SC Emotion C (hourly)	0.096	−0.095	0.024	0.148 *	0.130	0.171 *	0.016	0.170 *	−0.046	0.054	−0.079	−0.011	−0.070	−0.135	−0.105	−0.099	−0.042	−0.063	0.030	−0.144 *	−0.074	−0.040	−0.209 **	1		
25. SC Cognition Q (hourly)	−0.010	0.045	0.036	0.154 *	0.178 *	0.021	0.087	0.411 **	0.141 *	0.056	0.163 *	0.112	0.142 *	0.119	0.103	−0.043	0.048	0.144 *	0.104	0.054	0.144 *	0.102	0.232 **	−0.083	1	
26. SC Cognition C (hourly)	0.158 *	0.033	−0.006	0.218 **	0.230 **	0.166 *	0.031	0.265 **	−0.056	−0.036	−0.007	0	0.038	−0.099	0.097	−0.058	−0.055	−0.098	−0.070	−0.049	0.062	0.114	−0.193 **	0.180 *	0.056	1

Note. Variables no. 8–26 are all occurrences of mothers’ MST (hourly). In addition to gender, correlations were estimated using Spearman’s correlation. ToM1: unexpected location task; ToM2: unexpected content task; ToM3: appearance–reality task. * Indicates *p* < 0.05. ** Indicates *p* < 0.01.

## Data Availability

The data that support the findings of this study are available from the corresponding author upon reasonable request.
